# A Water-Soluble Inclusion Complex of Pedunculoside with the Polymer β-Cyclodextrin: A Novel Anti-Inflammation Agent with Low Toxicity

**DOI:** 10.1371/journal.pone.0101761

**Published:** 2014-07-11

**Authors:** Chang Liu, Wang Zhang, Hao Yang, Weidong Sun, Xiangdong Gong, Junxian Zhao, Yun Sun, Guowang Diao

**Affiliations:** 1 College of Medicine, Yangzhou University, Yangzhou, Jiangsu, P. R. China; 2 College of Chemistry and Chemical Engineering, Yangzhou University, Yangzhou, Jiangsu, P. R. China; 3 Chinese Medicine Hospital of Yangzhou City, Yangzhou, Jiangsu, P. R. China; National Institutes of Health, United States of America

## Abstract

More than 50% of new drug candidates in drug discovery are lipophilic and exhibit poor aqueous solubility, which results in poor bioavailability and a lack of dose proportionality. Here, we improved the solubility of pedunculoside (PE) by generating a water-soluble inclusion complex composed of PE and the polymer β-cyclodextrin (CDP). We characterized this novel complex by ^1^H NMR, FT-IR, UV-vis spectroscopy, powder X-ray diffractometry and thermogravimetric analysis. The ratio of β-cyclodextrin (β-CD) units in CDP to PE was determined to be 2∶1. The *K*_D_ value of the inclusion complex was determined to be 4.29×10^−3^ mol•L^−1^. In contrast to the low solubility of PE, the water-solubility of the PE–CDP complex was greatly enhanced. A preclinical toxicological study indicated that PE–CDP was well tolerated for a single administration. Importantly, the anti-inflammation potency of the PE–CDP complex was higher than that of PE. As a result, the formation of inclusion complexes by water-soluble CDP opens up possible aqueous applications of insoluble drug candidates in drug delivery.

## Introduction

Terpenoids represent a class of natural products that provide a wealth of opportunities to address many human-health issues [1]–[4]. Notably, many terpenoids are known for their anti-inflammatory properties [5]–[8]. Pedunculoside (PE) was extracted from bark of *Ilex rotunda Thunb*, which is a pentacyclic triterpenoid. Modern pharmacology showed that PE possesses hypolipidemic and anti-myocardial ischemia effects [9], [10]. Additionally, our previous data have demonstrated that PE can inhibit the inflammatory response induced by multiple irritant agents. However, PE and other pentacyclic triterpenes suffer from low oral bioavailability, critically limiting their clinical potential [11], [12].

Cyclodextrins (CDs) are cyclic oligosaccharides composed of six to eight glucose units linked by α-1,4-glucosidic bonds. Because of their hydrophobic internal cavity and hydrophilic external surface, their unique molecular structure can form supramolecular host–guest complexes with various hydrophobic molecules [13], [14]. Based on their distinct physical and chemical properties [15], CDs are used in various fields, including in biological medicine [16]–[19], foodstuffs [20], [21], and environmental protection [22]–[24]. The polymer β-cyclodextrin (CDP) was synthesized by the reaction of β-CD with a cross-linking agent, epichlorohydrin [25]. CDP is a highly water-soluble macromolecule that is well known to selectively form inclusion complexes [26], [27]. Our results have demonstrated that CDP can overcome the drawbacks of β-CD—such as poor water-solubility and the restriction of the single cavity size [27]–[29]. Ilexgenin A (IGA), which is structurally a pentacyclic triterpene, was extracted from the leaves of *Ilex hainanensis Merr*, has lipid-lowering effects on hyperlipidemia in mice and represents a novel lipid-lowering drug candidate. However, the application of IGA has been restricted due to its poor solubility. The water-soluble host CDP improved the physical and chemical properties of the IGA guest by forming an IGA–CDP inclusion complex. Moreover, IGA-CDP was more effectively for treating mice with hyperlipidemia because its water-solubility contributes to absorption of IGA-CDP *in vivo*, which improves its bioavailability [30]. All of these results indicated that CDP could be used as a universal solubilizer for pharmaceutical applications.

Here, for the first time, we used CDP as a solubilizing agent to improve the water-solubility of PE, and we characterized the formation of a water-soluble inclusion complex of PE and CDP. The method provides a convenient and efficient approach for obtaining PE with high water solubility. Additionally, we evaluated the safety of oral administration of PE-CDP. Our results showed that PE–CDP displays minimal toxic effects when administered to mice, which makes it possible to perform further toxicological and pharmacological studies in humans. Furthermore, we compared the anti-inflammatory effects of PE and PE–CDP in the murine models of inflammation. Surprisingly, PE–CDP was more effectively for treating skin inflammation, because its water solubility contributes the absorption of PE–CDP *in vivo*.

## Materials and Methods

### Chemicals, material and reagents

Pedunculoside (purity >99%.) was obtained from Anshi Pharmaceutical Co., Ltd (Zhongshan, China), β-CD, epichlorohydrin, ethylene glycol and other reagents were all of analytical purity and purchased from Shanghai Chemical Reagents Company. Doubly distilled and sterilized water was obtained from a Milli-Q UV system (Millipore).

### Instruments

The FTIR spectrum was measured using a Tensor spectrophotometer (Bruker, Germany). The UV-Vis spectra were recorded on a UV-2550 double-beam spectrophotometer (Shimazu, Japan) equipped with a stoppered quartz cell with a 1.0-cm optical path length. The ^1^H NMR spectrum was measured on a 600-MHz Bruker spectrometer (Bruker, Germany) at 303.1 K in deuterium oxide. Powder X-ray diffraction spectrum was measured by a D8 super speed X-ray instrument (Bruker, Germany) with Cu Kα radiation, *λ* = 1.542 Å. Thermogravimetric analysis (TGA) was performed on a thermogravimetric analyzer (PerkinElmer, Pyris 1 TGA) with approximately 10-mg samples, which were heated from room temperature to 600°C at a rate of 10°C min^−1^ under nitrogen atmosphere. Hematological parameters were determined from blood collected into tubes containing trisodium citrate and were analyzed using an automatic veterinary hematology analyzer (Hemavet 950, USA). A slicer (Lieca, Germany) was used to cut 4-µm slices, and the sections were stained with H&E and examined by light microscopy (Nikon, Japan).

### Preparation of the PE–CDP inclusion complex

We prepared CDP by a similar method to that reported in [27]. Briefly, CDP was synthesized by cross-linking β-CD with EP under strongly alkaline conditions (33 wt% NaOH). The molar ratio of β-CD/EP was 1∶7.

The preparation of the inclusion complex of PE with CDP was as follows: first, we dissolved 0.5 g CDP and 0.2 g PE in 50 ml water with stirring for at least 12 h at room temperature. Second, a white solution containing the inclusion complex was obtained after filtering the residual PE. Third, PE–CDP was obtained by pressure distillation, and it was dried in a vacuum oven at 60°C for 24 h.

### Aqueous solubility of PE–CDP

The solubility of PE–CDP in water was measured at 25°C according to reference [28]. To ensure that the solution was saturated with PE–CDP, excess inclusion complex was added into 5 ml water, which was mechanically shaken for 2 h at 25°C. Then, the remaining solid in the solution was filtered off using a 0.45-µm Cameo Nylon syringe filter. The solubility of PE–CDP in water could be calculated from the difference between the initial amount and the residual amount of PE–CDP. Also, the saturated concentration of PE–CDP in water was determined by UV–Vis spectrophotometry. All of the measurements were repeated three times.

### Acute-dose toxicity study

In the present study, ICR mice (6–8 week old) were obtained from Yangzhou University Comparative Medicine Center (License No: SCXK (Su) 20120009) and kept in a room on a 12 h light/dark cycle and at a temperature of 23–25°C and 50±5% humidity. All animal experiments performed in this study were approved by the Institutional Animal Care and Use Committee of Yangzhou University.

The study design was evaluated following the Organization for Economic Cooperation and Development Guide lines for the Testing of Chemicals no. 420 (OECD, 2001) with slight modifications [30]. The animals were fasted prior to conducting the experiment (only food but not water was withheld overnight). This study was conducted over 15 days (days 0–14). Briefly, the mice were divided into three groups of 20 each (ten females and ten males). On day 0, the group 1 (control) mice were intragastrically administered a 0.5% carboxymethyl cellulose (CMC) sodium aqueous solution. The group 2 mice were intragastrically administered PE at a dose of 2000 mg•kg^−1^ body weight. The PE was suspended in a 0.5% CMC sodium aqueous solution. The group 3 mice were intragastrically administered PE–CDP at a dose of 8985 mg•kg^−1^ (PE content of 2000 mg•kg^−1^) body weight. The mice were observed individually for signs of acute toxicity and behavioral changes for 4 h post dosing and at least once daily for 14 days. The food and drink consumption and body weight were recorded daily. Histopathological and hematological examinations were performed for all mice on day 14.

### Anti-inflammation activities of PE–CDP

#### Drug administration

After one week of adaptive feeding, the forty male ICR mice were randomly divided into five groups (n = 8). In the model group, the mice were administered with equal volumes of a 0.5% CMC aqueous solution. In the positive control group, the mice were administered aspirin at a dose of 100 mg•kg^−1^•d^−1^. In the test-drug group 1, the mice were administered PE at a dose of 20 mg•kg^−1^•d^−1^. In the test-drug group 2, the mice were administered PE–CDP at doses of 45 or 90 mg•kg^−1^•d^−1^ (PE content of 10 or 20 mg•kg^−1^•d^−1^, respectively).

#### Dimethyl benzene-induced mice ear edema

Ear edema was induced in the right ear of mice by the topical application of dimethyl benzene, as previously described with a slight modification [31]. The previous method was conducted over seven days. However, in our study, the experiment was conducted over six days (days 0–5). On days 0–4, the mice were orally administered the drugs once per day. On day 5, the mice were orally administered the drugs after 30 min: additionally, the right ear of each mouse was treated with dimethyl benzene (40 µL) on both ear surfaces, and the left ear was used as a control. The mice were sacrificed, and the right-ear biopsies were obtained with a punch (6-mm diameter) after 15 min.

The edema weight and inhibition percentage was calculated using the following equation:

Edema weight  =  weight of the right ear–weight of the left ear

Inhibition %  =  (edema weight of control group–edema weight of treated group)/edema weight of control ×100%.

#### Croton oil-induced mice ear edema

Croton oil-induced mice-ear edema was determined according to a previously described method with minor modifications [32]. This study was conducted over nine days (days 0–8). The previous method showed that 5% (v/v) croton oil in acetone (20 µL) was applied on alternate days. In our study, on days 0–3, 5% (v/v) croton oil in acetone (20 µL) was applied with a micropipette onto the right ears of the mice, and acetone onto the left ears. The ear edema was evaluated daily by measuring the ear thickness. On days 4–7, the mice were orally administered the drugs once per day. On day 8, the mice were sacrificed, and the right ear biopsies were obtained with a punch (6-mm diameter). The inhibition ratio of the ear swelling was calculated as described above.

### Statistical analysis

Statistical analyses were conducted using SPSS ver. 11.5(SPSS, Chicago, IL, USA). The results are shown as the mean ± S.D., and the significance of the differences was determined using the two-way analysis of variance (ANOVA) followed by the Student–Newman–Keuls multiple-comparison test (SNK) as post hoc. The differences were consider significant if *p*<0.05.

## Results and Discussion

### Characterization of PE–CDP

**Figure 1:**
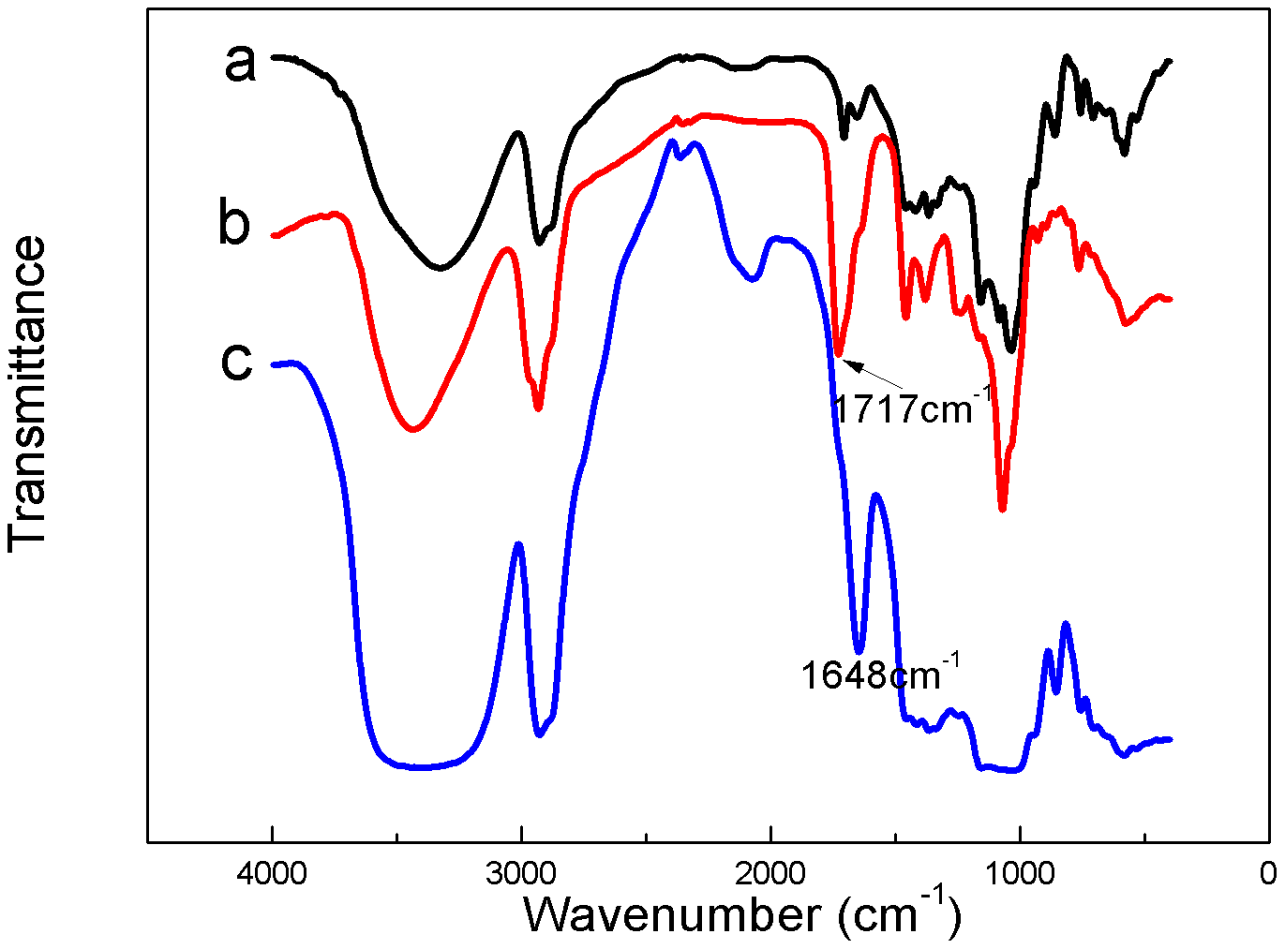
FTIR spectra of (a) CDP, (b) PE, and (c) PE-CDP.

Fig. 1 showed the FTIR spectra of (a) CDP, (b) PE, and (c) PE–CDP. A typical IR spectrum for CDP was presented in Fig. 1(a), showing features such as the coupled C-O-C stretching vibrations at ∼1155 cm^−1^, the coupled C-O/C-C stretching vibrations at ∼1040 cm^−1^, the CH_2_ stretching vibrations at ∼2930 cm^−1^, and the OH stretching vibrations at ∼3335 cm^−1^. Fig. 1(b) showed the typical absorptions of PE, such as the carbonyl and aromatic stretching vibrations at 1717 cm^−1^, which is similar to those of pentacyclic triterpenoids [29]. As shown in Fig. 1(c), we found that the FTIR spectrum of PE–CDP exhibited the typical absorption features of CDP and PE. Compared with PE, the peak of the carbonyl and aromatic stretching vibrations shifted to 1648 cm^−1^, and the peak strength became lower than that of PE. In addition, the stretching-vibration absorption of hydroxyl at approximately 3400 cm^−1^ exhibited a slight blue shift compared to that of CDP. The results indicated the formation of an inclusion complex of PE and CDP.

**Figure 2:**
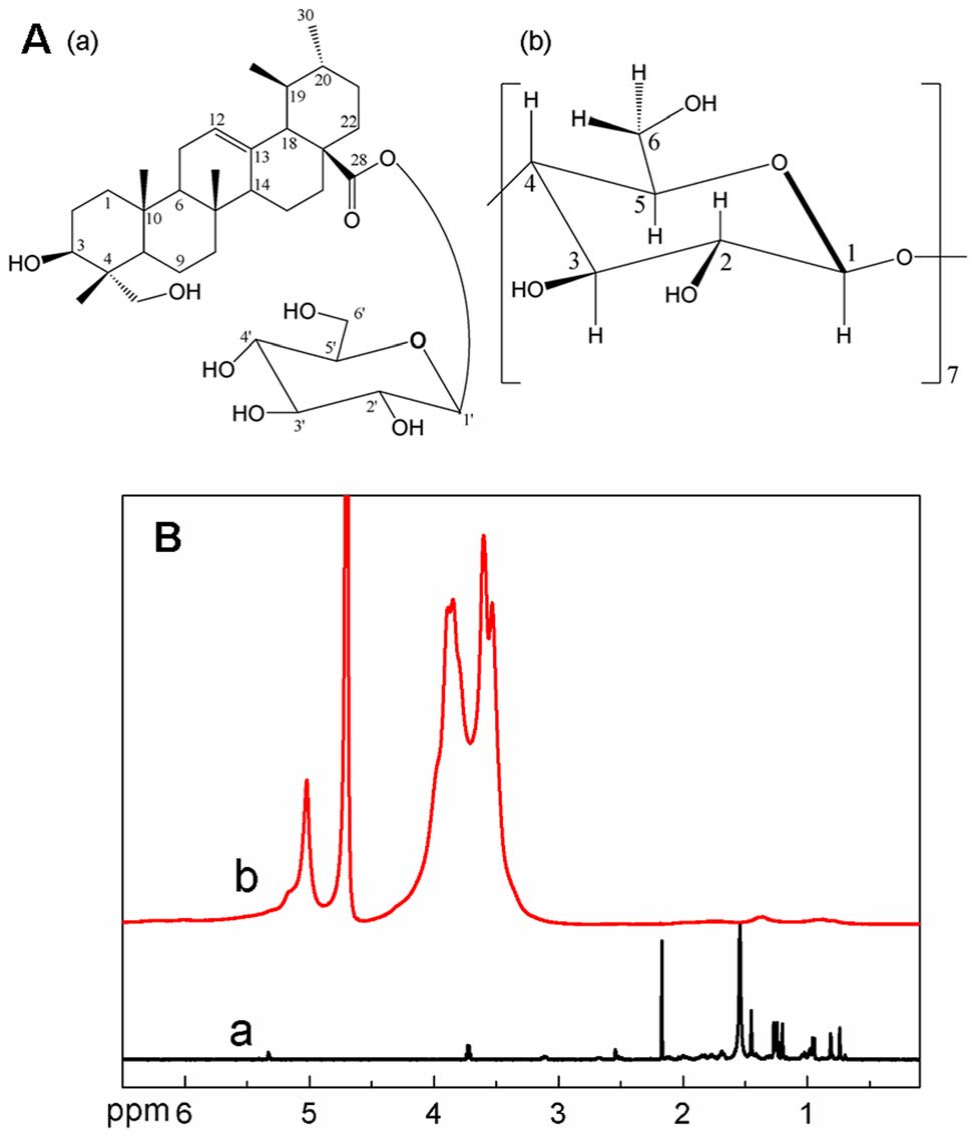
A Chemical structure of (a) PE and (b) the CD unit in CDP. **B** ^1^H NMR spectra of (a) PE and (b) PE-CDP.

The ^1^H NMR spectrum provides direct evidence for the formation of an inclusion complex [33]. The chemical structure of the (a) PE and (b) β-CD units in CDP was shown in Fig. 2A. To study the possible modes of PE–CDP, we compared the ^1^H NMR spectra of PE in the absence and presence of CDP in Fig. 2B. The ^1^H resonance of CDP was assigned according to the reported method [25], [27]. The typical ^1^H NMR spectrum of PE was shown in Fig. 2B (a), which was consistent with that previously reported [34]. The majorities of the PE chemical shifts were from 0.5 to 2 ppm and were distinct from those of the CDP protons. After the interaction of PE with CDP, the ^1^H NMR spectrum in Fig. 2B (b) confirmed the existence of PE in the inclusion complex. The H-12 proton of PE shifted from 5.33 to 6.01 ppm, as listed in the Electronic Supplementary Information (ESI, Table S1). The results indicated that the PE guest penetrated into the β-CD cavities in CDP to form an inclusion complex of PE–CDP. The ratio of PE to β-CD units in the polymer inclusion complex was determined by the peak area of protons between PE (H-12) and β-CD unit (H1) in CDP. The measured number of the protons located at different positions is listed in Table S2. The stoichiometries (2.05/1 molar ratio) of both the β-CD units of the polymer and PE were confirmed by ^1^H NMR spectroscopy.

The inclusion complex was also confirmed by X-ray diffractometry [34]. Fig. S1 in the ESI shows the powder X-ray diffraction patterns of (a) PE and (b) PE–CDP. In Fig. S1 (a), sharp PE peaks at diffraction angles from 5° to 25° were observed, confirming that PE existed as a crystalline material. In Fig. S1(b), only a broad peak at 2*θ* = 13.8° appeared on the curve of PE–CDP, and the X-ray partten of PE disappeared for PE–CDP compared with that shown in Fig. S1(b). The results proved that PE-CDP existed in an amorphous state by the supramolecular interaction of CDP and PE.

The thermal stability of the inclusion complex was determined by thermogravimetric (TG) analysis in the temperature range from 30 to 800°C. Fig. S2 in the ESI showed the TG curves of the inclusion complexes of PE and PE–CDP, respectively. PE was thermally stable and decomposed above 300°C. In Fig. S2, the inclusion complex of PE–CDP began to dissociate at 280°C, because the temperature was similar to the melting point of the CD component. A loss of weight was also observed at 452–796°C, which corresponded to the thermal decomposition of PE in PE–CDP, indicating that the thermal stability of PE in PE–CDP was better than that of neat PE, due to the strong supramolecular interaction between PE and PE-CDP. Moreover, the amount of PE in the inclusion complex was determined to be 14 wt%.

### UV-Vis spectra and dissociation constant of the inclusion complex

Fig. S3 in the ESI shows the UV-Vis absorption spectra of PE (a) and PE–CDP (b) in water. Because of the poor water solubility of PE, no absorption was observed for pure PE in the range of 200 to 500 nm, as shown in Fig. S3 (a). However, there was only one peak observed at 266 nm in Fig. S3 (b). The PE structure of the pentacyclic triterpenoid resulted in a strong absorption throughout the UV spectrum of PE–CDP with a peak at 266 nm, which was consistent with results from the literature [35]. Therefore, the spectral profile of PE was markedly affected by the formation of an inclusion complex with CDP. It was clear that the solubility of PE–CDP was much higher than that of PE. These results indicated that CDP formed an inclusion complex with PE, which improved the aqueous solubility of PE.

**Figure 3:**
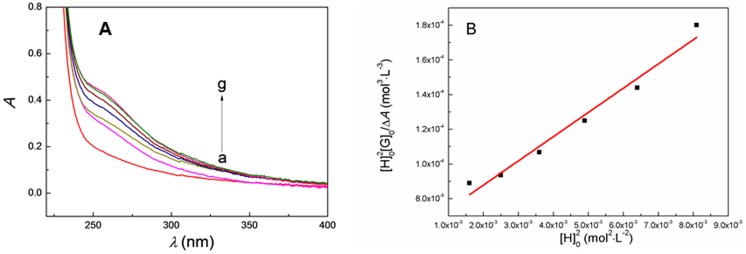
A At 25°C, the UV-Vis spectra of 4 mM PE in ethylene glycol with various concentrations of the β-CD unit in β-CDP (mM): a) 0, b) 40, c) 50, d) 60, e) 70, f) 80 and g) 90. **B** The plot of [H]2 0[G]_0_/Δ*A* vs. [H]2 0.

Because of the poor water solubility of PE, ethylene glycol, a suitable solvent for both PE and CDP, was used to study the dissociation constant of the inclusion complex. The supramolecular interaction between PE and CDP in ethylene glycol was confirmed by the UV-Vis spectrum, as shown in Fig. 3A. It was found that the peak position of PE–CDP was independent of the addition of CDP. However, as the concentration of CDP rose, the peak intensity increased. Assuming that the ratio of PE to the β-CD units of the CDP was 1∶2, the formation of the inclusion complex could be described by the Benesi–Hildebrand method [27], [36]:
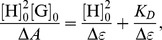
where H represented the host, the β-CD unit in the polymer, and G was the guest, PE. *K*_D_ was the dissociation constant of PE–CDP. The initial concentrations of H and G were [H]_0_ and [G]_0_, respectively, and [H]_0_≫[G]_0_. 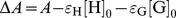, 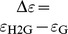, *A* was the UV-Vis absorbance, and ε_H_, ε_G_, and ε_H2G_ were molar -absorption coefficients of H, G, and PE–CDP, respectively.

Plotting 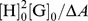 versus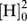, a straight line was obtained, as shown in Fig. 3B. The assumption of a 1∶2 ratio of PE to the β-CD units in the CDP was proven, because of the linear relationship between 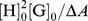 and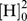. According to the slope and the intercept of the line, *K*_D_ of the inclusion complex was determined to be 4.29×10^−3^ mol•L^−1^. The result was similar to that of the pentacyclic triterpenoid ilexgenin A in our previous study [28].

### Acute-dose toxicity study of PE–CDP

Safety is a primary concern when considering new excipients intended for use in pharmaceutical formulations [35]. Although CDs have many advantages as candidates for novel drug carriers, few studies evaluating safety evaluation have been performed for chemically modified CDs, such as CDP [36], [37]. Here, the administration of PE–CDP at an 8985-mg•kg^−1^ dose resulted in no deaths in mice. Furthermore, throughout the experimental period, all animals appeared to be healthy, with no visible signs of pain, distress, or discomfort. Conversely, there were no significantly differences in the organ weight between the treatment groups and the control group (ESI, Table S3). Additionally, all mice remained active and exhibited good weight gain (ESI, Fig. S4).

In the hematology parameters, the oral administration of PE–CDP to mice did not remarkably affect the blood hematologic parameters, because most parameters are within in the physiological range (Table 1). However, in females, a statistically significant decrease in the red blood cells concentration was observed in the PE–CDP group in comparison with those of the control group (*p*<0.01). Additionally, the red blood cell specific volume and the mean corpuscular hemoglobin in the PE–CDP group were significantly increased (*p*<0.05). It is known that native CDs induce shape changes and membrane invagination in erythrocytes [38], [39], which may lead into an increase in red blood cells concentration, the red blood cell specific volume and the mean corpuscular hemoglobin in mice.

**Table 1:** Hematological parameters in mice treated with PE (2000 mg•kg^−1^) or PE-CDP (8985 mg•kg^−1^) after 14 days.

	Experiment groups		
***Male***	Control (0.5% CMC)	PE (2000 mg•kg^−1^)	PE-CDP (8985 mg•kg^−1^)
**RBC(10^12^.L^−1^)**	9.91±0.22	10.36±0.77	10.28±0.58
**Hb (g/L)**	138±2	140±8.8	142±5.5
**HCT (%)**	62.4±1.8	65.1±4.3	64.2±2.3
**MCV (fL)**	63±1	62.9±1	62.6±2.7
**MCH (Pg)**	13.9±0.34	13.5±0.4	13.8±0.54
**MCHC (g/L)**	221±7.1	215±4.9	221±5.3
**RDW (%)**	17.1±0.60	17±0.85	17±0.91
**WBC(10^9^.L^−1^)**	5.74±1.1	8.02±4.94	7.3±1.87
**LY (%)**	35.8±12.1	37.7±6	42±7.1
**NE (%)**	52.9±13.4	50.1±6.3	43.7±6.3
**MO (%)**	10.6±8	11.4±3.6	13.4±2.9
**EOS (%)**	0.57±0.29	0.3±0.19	0.24±0.14
**BASO (%)**	0.06±0.02	0.05±0.03	0.09±0.04
**PL (10^9^.L^−1^)**	976±79	889±147	1078±162
**MPV (fL)**	5.1±0.2	4.9±0.26	5.12±0.36
***Female***			
**RBC(10^12^.L^−1^)**	9.87±0.25	10.26±0.82	10.55±0.17**
**Hb (g/L)**	144±7.7	147±7.5	141±15
**HCT (%)**	60.3±3.8	65.1±3.2	65±0.9*
**MCV (fL)**	61.1±2.7	63.7±3.1	61.7±1.8
**MCH (Pg)**	14.6±0.61	14.3±0.65	13.9±0.29*
**MCHC (g/L)**	240±19	225±1.5	226±3.6
**RDW (%)**	17±0.5	16.6±0.6	17±0.5
**WBC(10^9^.L^−1^)**	6.6±2	9.4±0.7*	6.7±1.9
**LY (%)**	28.3±13.6	44.1±7	38.1±6.2
**NE (%)**	46.6±10.7	36±3.5*	46.5±5.2
**MO (%)**	11.2±8	18±4	14±3
**EOS (%)**	1.33±0.7	1±0.4	2.1±1.4
**BASO (%)**	0.16±0.07	0.07±0.04	0.13±0.05
**PL (10^9^.L^−1^)**	907±148	955±204	874±93
**MPV (fL)**	4.8±0.23	4.73±0.17	4.98±0.18

Data are expressed as the means ± S.D. (n = 10).

*p<0.05,

**p<0.01, verus the control group.

Histopathology is an important factor for determining the treatment performance and effects, especially the negative effects [40]. As shown in Fig. S5, the histopathological evaluation of tissues showed no evidence of toxic effects for 8985-mg•kg^−1^ PE–CDP administration. In the heart, the myocardium and myocardial interstitial tissue were in normal. Additionally, no myocardial fibrosis or inflammatory cell infiltration could be observed in the PE–CDP treatment group. In the liver hepatic lobule, the sinusoidal, plate and hepatic cell structure were normal. In the spleens, the cells around the central arteriole did not proliferate. The lung histology of the PE–CDP-treated group showed normal structure, and in the lung tissues, there was no infiltration of inflammatory cells with edema and the proliferation of collagen. In the kidneys, the structure of the renal cortex and the medulla was normal, and in the renal corpuscles, there was no hyperaemia or exudation.

In summary, the acute oral administration of PE–CDP at 8985 mg•kg^−1^ to male and female mice caused no animal deaths. Therefore, it was impossible to determine the LD_50_. Additionally, there were no obvious signs of toxicity, including abnormal changes in behavior and body weight for 14 days of monitoring. Moreover, no changes were observed in the macroscopic and histopathological analysis. These observations are consistent with the findings of the most recent investigations into CDP and CDs conjugated to other delivery vehicles, namely nanoparticles and liposomes [41], [42]. Additionally, the results provided an advantageous condition for its anti-inflammation effects.

### Anti-inflammation activities of PE–CDP

The application of mice models for ear edema induced by various irritant agents has been widely used to identify the probable anti-inflammatory effect of the natural products [22], [32]. Mice-ear edema is also often induced by the application of croton oil or dimethyl benzene. The application of dimethyl benzene application provides data regarding the anti-inflammation activity in an acute inflammatory process, whereas multiple applications of croton oils can be used to evaluate the anti-inflammation activity in an established chronic-inflammatory process [43], [44].

**Figure 4:**
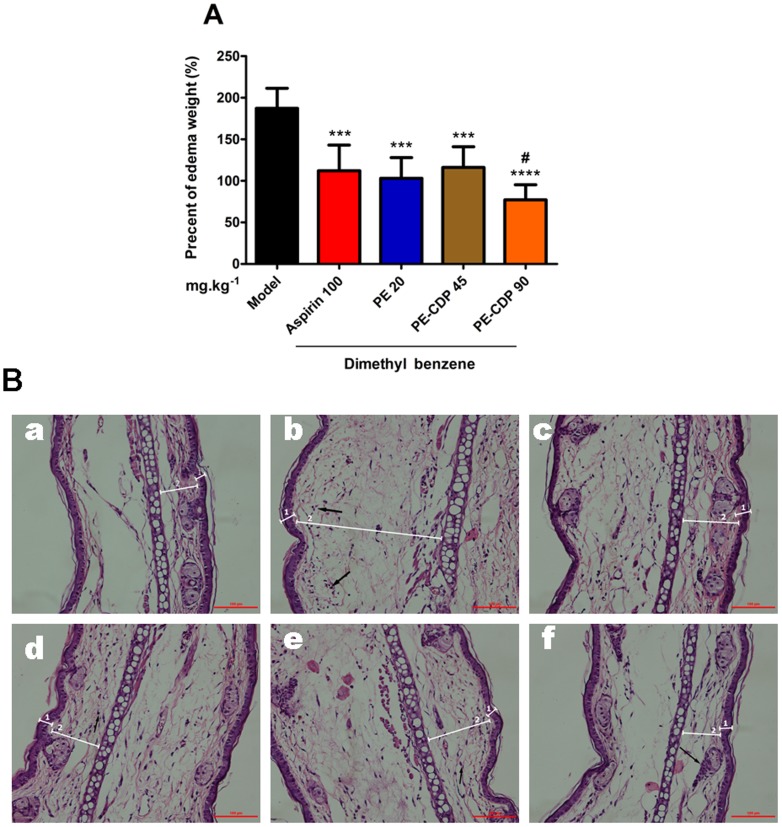
A The effect of PE-CDP on dimethyl benzene application-induced ear edema. (n = 8). **B** Histological examinations of mice sensitized with the topical application of dimethyl benzene, stained with hematoxylin–eosin and examined under light microscopy. Treatments: non-inflamed (a), inflamed ear (b), Aspirin: 100 mg•kg^−1^•d^−1^ (c), PE 20 mg•kg^−1^•d^−1^ (d), PE-CDP 45 mg•kg^−1^•d^−1^ (e) and PE-CDP 90 mg•kg^−1^•d^−1^ (f). The numbers 1 and 2 indicate the epidermis and dermis, respectively.

To examine the possible anti-inflammatory effect of PE–CDP, the mice were subjected to the topical application of dimethyl benzene on the ear to model skin inflammation. The local application of dimethyl benzene stimulates a rapid inflammatory response [45], contributing an immediate vasodilation and erythema in the right ear when compared with the left ear. As shown in Fig. 4A, after 30 minutes of administering PE–CDP or PE intragastrically, the weight of the right ear decreased significantly. Treatment with 90 mg•kg^−1^•d^−1^ PE–CDP and 20 mg•kg^−1^•d^−1^ PE significantly reduced the size of the edema by 58.8% and 44.9%, respectively. Importantly, the results showed that the application of 90 mg•kg^−1^•d^−1^ PE–CDP caused a significant reduction in the ear edema when compared to the 20 mg.kg^−1^.d^−1^ PE-treated group (*p*<0.05). Notably, treatment with 90 mg•kg^−1^•d^−1^ PE–CDP was similar to that of the treatment with aspirin 100 mg.kg^−1^.d^−1^, which is the positive control group in the study.

The histological analysis revealed that dimethyl benzene application leads to a significant increase in the dermis thickness, which was accompanied by a loosening of the connective tissue and a disorganization of the fibers from the extracellular matrix (Fig. 4B: b). In addition, the mice treated with PE or PE–CDP exhibited a lower dermis thickness (Fig. 4B: d–f), compared to that of the untreated inflamed ear. Importantly, the treatment with 90 mg•kg^−1^•d^−1^ of PE–CDP improved the degree of ear edema more effectively than 20 mg•kg^−1^•d^−1^ of PE.

**Figure 5:**
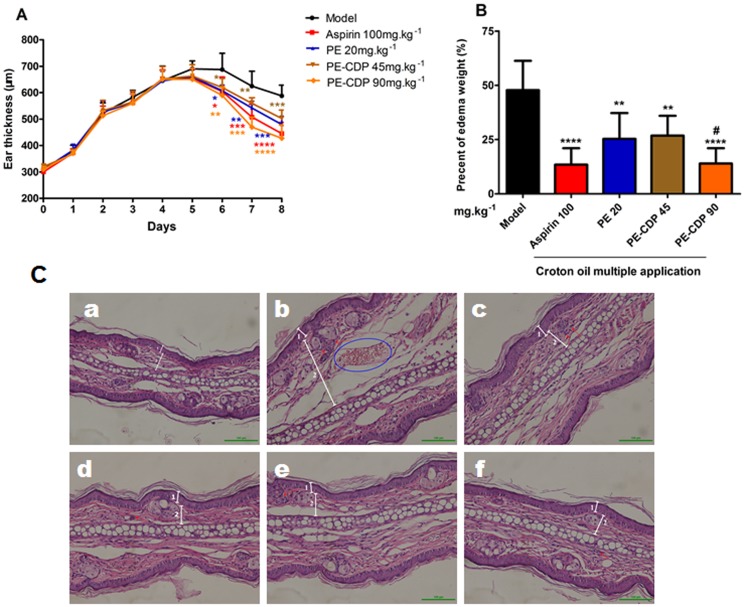
Effect of PE-CDP on croton oil multiple application-induced ear edema. (**A**) shows the time response curve of the effect from days 0 to 8. Croton oil in acetone was applied on days 0–4. The thickness of the ear was measured daily, using a digital caliper. On days 5–8, the ear of the animals received a 0.5% CMC aqueous solution, aspirin, PE or PE-CDP. (**B**) shows the percentage of edema weight for each group on day 8. *P<0.05, **P<0.01, ***P<0.001 and ****P<0.0001 compared to the 0.5% CMC aqueous solution-treated group, ^#^P<0.05 compared to the PE 20 mg•kg^−1^•d^−1^ group (n = 8). **C** Histological examinations of mice sensitized with multiple applications of croton oil, stained with hematoxylin–eosin and examined under light microscopy. Treatments: non-inflamed (a), inflamed ear (b), aspirin: 100 mg•kg^−1^•d^−1^ (c), PE 20 mg•kg^−1^•d^−1^ (d), PE-CDP 45 mg•kg^−1^•d^−1^ (e) and PE-CDP 90 mg•kg^−1^•d^−1^ (f). The numbers 1 and 2 indicate the epidermis and dermis, respectively. The arrows indicate inflammatory cells in the dermis. The blue areas indicate red cells.

We next assessed the effect of PE–CDP on mouse-ear edema was induced by croton oil. As expected, repeated croton oil application is associated with an increase in the ear weight, intense neutrophilical infiltration and macrophages migration. As shown in Fig. 5A, PE–CDP improved the degree of ear edema more rapidly and effective than PE. Additionally, the application of 90 mg•kg^−1^•d^−1^ of PE–CDP on day 6 caused a significant reduction in the ear thickness when compared to the 0.5% CMC aqueous solution-treated group (on day 6, *p*<0.01; on day 7, *p*<0.001; and day 8, *p*<0.0001.). The results observed on day 8 were confirmed by the evaluation of the edema weight (Fig. 5B). Treatment with 90 mg•kg^−1^•d^−1^ PE–CDP and 20 mg•kg^−1^•d^−1^ PE significantly reduced the sizes of the edemas by 70.1% and 47%, respectively. Additionally, the effect of the treatment with 90 mg•kg^−1^•d^−1^ of PE–CDP on the ear edema induced by croton oil was similar to that of the treatment with aspirin 100 mg.kg^−1^.d^−1^.

Fig. 5C showed that multiple applications of croton oil lead into a significant increase in the dermis thickness, which was accompanied by disorganization of the fibers from the extracellular matrix and the infiltration of inflammatory cells. The mice treated with PE or PE–CDP revealed a reduction in the infiltration of inflammatory cells (Fig. 5C: d–f), compared to the untreated inflamed ear. Moreover, the treatment with 90 mg•kg^−1^•d^−1^ of PE–CDP improved the degree of ear edema more effectively than 20 mg•kg^−1^•d^−1^ of PE. This result is consistent with the effect of PE–CDP on mouse-ear edema that was induced by dimethyl benzene.

In summary, both PE and PE–CDP exhibited anti-inflammatory effects induced by dimethyl benzene and croton oil on mouse-ear edema. Furthermore, treatment with 90 mg•kg^−1^•d^−1^ of PE–CDP improved the degree of ear edema more effectively than 20 mg•kg^−1^•d^−1^ of PE in the murine models of inflammation. It was reported that CDs can enhance the absorptive capacity of poorly water-soluble drugs in gut epithelial cells [18], [46]. Thus, PE–CDP acted more effectively on mice-ear edema, possibly because CDP contributes to the absorption of PE–CDP *in vivo*.

## Conclusions

We developed a convenient and efficient method to solve the low-solubility issue of PE by generating a PE–CDP complex that exhibited high solubility and bioavailability. This noncovalent modification by the CDP largely preserved the integrity of PE, which is critical for medicinal applications. Additionally, we evaluated the safety of the oral administration of PE–CDP in mice and the anti-inflammatory effect of PE–CDP for irritant agent-induced ear inflammation in mice. The results revealed that PE–CDP not only has extreme low toxicity, but it also acted more effectively on mice-ear edema, possibly because the water solubility contributes to the absorption of PE–CDP *in vivo*. Furthermore, the formation of inclusion complexes by water-soluble CDP provides a novel solution for possible aqueous applications of insoluble products in drug delivery.

## Supporting Information

Figure S1 **The XRD spectra of (a) PE and (b) PE–CDP.**
(DOC)

Figure S2 **TG curves of (a) PE and (b) PE–CDP.**
(DOC)

Figure S3 **UV spectra of (a) PE–CDP and (b) PE in an aqueous solution of pH = 7.0 at 25°C.**
(DOC)

Figure S4 **Body-weight, food-intake and water-intake curves for mice administration with a single dose of PE (2000 mg•kg^−1^) or PE–CDP (8985 mg•kg^−1^) over 14 days (n = 10).**
(DOC)

Figure S5 **Histological examinations including the heart (A), liver (B), spleen (C), lung (D) and kidney (E) sections in mice after administration with a single dose of PE (2000 mg•kg^−1^) or PE–CDP (8985 mg•kg^−1^).**
(DOC)

Table S1 **Chemical shift** ***δ*** **(H-21) and Δ** ***δ*** **of protons of PE, PE–CDP.**
(DOC)

Table S2 **The number of the protons determined by ^1^H NMR in** **Fig. 3** **.**
(DOC)

Table S3 **Final body weight and organ weight in mice treated with PE (2000 mg•kg^−1^) or PE–CDP (8985 mg•kg^−1^) after 14 days.**
(DOC)
