# Supplementary figures and images for: A Water-Soluble Inclusion Complex of Pedunculoside with the Polymer β-Cyclodextrin: A Novel Anti-Inflammation Agent with Low Toxicity
Source: PLoS One. 2014 Jul 11;9(7):e101761. doi: 10.1371/journal.pone.0101761 (PMC4094462; doi:10.1371/journal.pone.0101761)

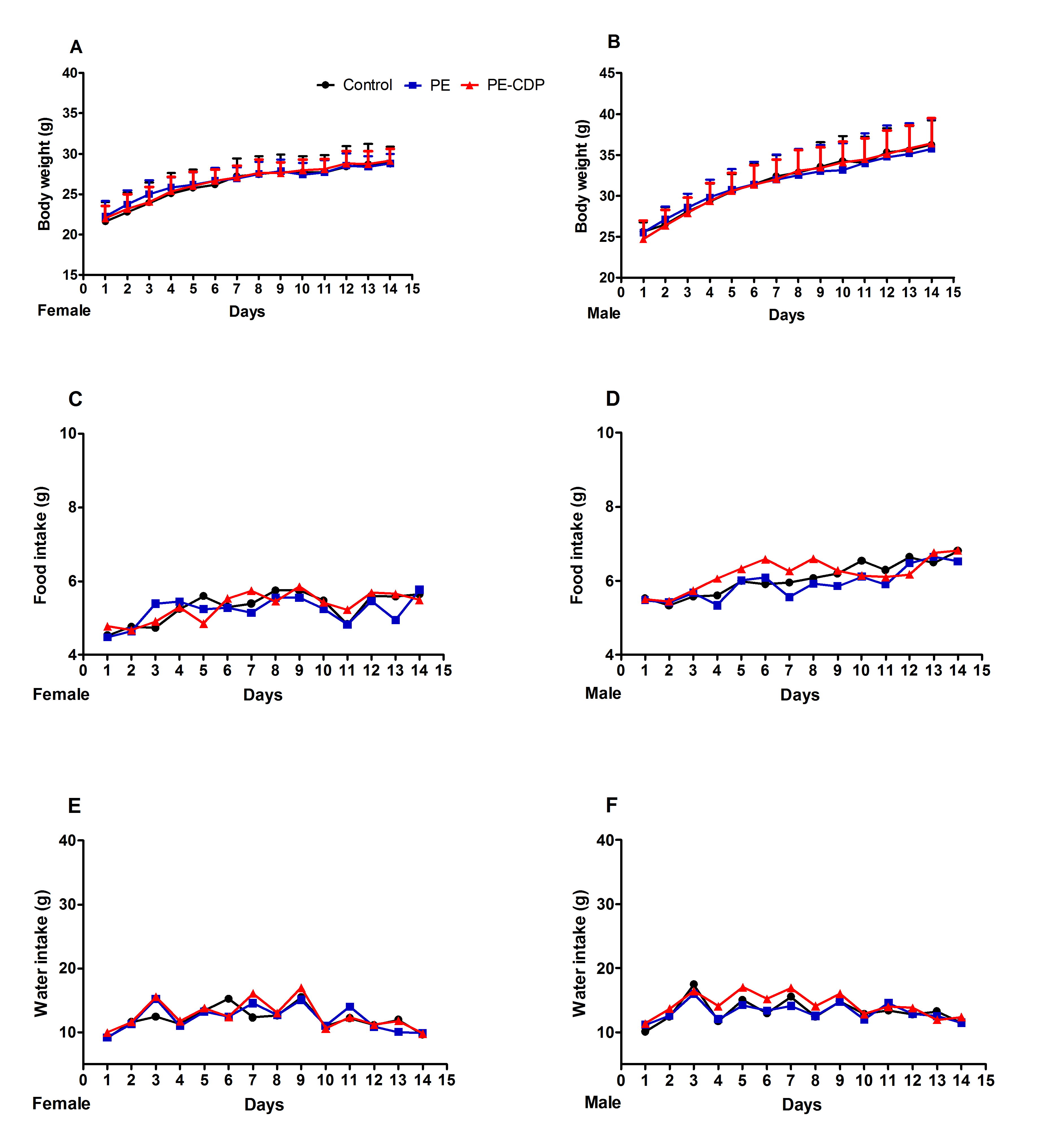

Supplement: Figure S4 — Body-weight, food-intake and water-intake curves for mice administration with a single dose of PE (2000 mg•kg−1) or PE–CDP (8985 mg•kg−1) over 14 days (n = 10). (DOC) [file pone.0101761.s004.doc]

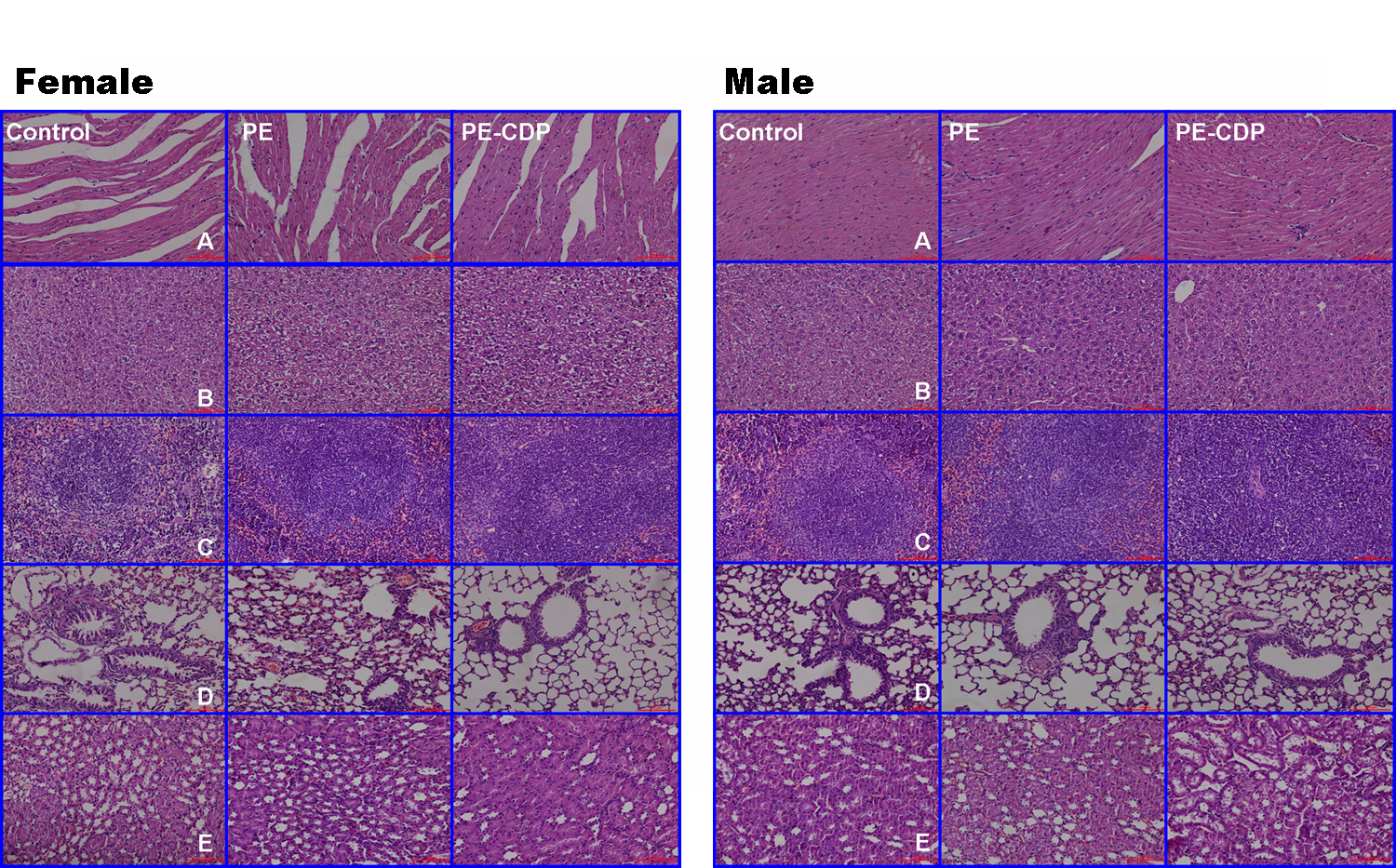

Supplement: Figure S5 — Histological examinations including the heart (A), liver (B), spleen (C), lung (D) and kidney (E) sections in mice after administration with a single dose of PE (2000 mg•kg−1) or PE–CDP (8985 mg•kg−1). (DOC) [file pone.0101761.s005.doc]
