# Supplementary material for: A Water-Soluble Inclusion Complex of Pedunculoside with the Polymer β-Cyclodextrin: A Novel Anti-Inflammation Agent with Low Toxicity
Source: PLoS One. 2014 Jul 11;9(7):e101761. doi: 10.1371/journal.pone.0101761 (PMC4094462; doi:10.1371/journal.pone.0101761)
Supplement: Table S1 — Chemical shift δ (H-21) and Δ δ of protons of PE, PE–CDP. (DOC) [file pone.0101761.s006.doc]

**Table S1 Chemical shift *δ* (H-21) and Δ*δ* of protons of PE, PE-CDP**

|  | H_21_ |
| --- | --- |
| PE | 5.33 |
| PE-CDP | 6.01 |
| Δ*δ* | 0.68 |
