# Supplementary material for: A Water-Soluble Inclusion Complex of Pedunculoside with the Polymer β-Cyclodextrin: A Novel Anti-Inflammation Agent with Low Toxicity
Source: PLoS One. 2014 Jul 11;9(7):e101761. doi: 10.1371/journal.pone.0101761 (PMC4094462; doi:10.1371/journal.pone.0101761)
Supplement: Table S2 — The number of the protons determined by 1H NMR in Fig. 3 . (DOC) [file pone.0101761.s007.doc]

**Table S2 The number of the protons determined by ^1^H NMR in Fig. 3.**

|  | H1 | H_21_ | CD/PE^a^ |
| --- | --- | --- | --- |
| PE -CDP | 14.32 | 1 | 2.05/1 |

^a^ In molar ratio
