# Supplementary material for: A Water-Soluble Inclusion Complex of Pedunculoside with the Polymer β-Cyclodextrin: A Novel Anti-Inflammation Agent with Low Toxicity
Source: PLoS One. 2014 Jul 11;9(7):e101761. doi: 10.1371/journal.pone.0101761 (PMC4094462; doi:10.1371/journal.pone.0101761)
Supplement: Table S3 — Final body weight and organ weight in mice treated with PE (2000 mg•kg−1) or PE–CDP (8985 mg•kg−1) after 14 days. (DOC) [file pone.0101761.s008.doc]

**Table S3.**

Final body weight and organ weight in mice treated with PE (2000 mg∙kg^−1^) or PE-CDP (8985 mg∙kg^−1^) after 14 days.

| **Weight (g)** | **Experiment groups** | | |
| --- | --- | --- | --- |
| **Male** | **Control (0.5% CMC)** | **PE (2000 mg∙kg^−1^)** | **PE-CDP (8985 mg∙kg^−1^)** |
| **Body** | 37.2±2.89 | 36.3±3.97 | 37.0±3.09 |
| **Heart** | 0.20±0.05 | 0.20±0.05 | 0.18±0.04 |
| **Liver** | 2.18±0.53 | 2.22±0.46 | 2.27±0.48 |
| **Spleen** | 0.15±0.04 | 0.16±0.05 | 0.14±0.04 |
| **Lung** | 0.22±0.04 | 0.20±0.04 | 0.22±0.04 |
| **Kidney** | 0.53±0.13 | 0.58±0.11 | 0.50±0.12 |
| **Female** |  | | |
| **Body** | 28.9±2.2 | 28.9±1.32 | 29.4±1.8 |
| **Heart** | 0.18±0.03 | 0.16±0.02 | 0.17±0.0003 |
| **Liver** | 1.76±0.17 | 1.79±0.18 | 1.76±0.30 |
| **Spleen** | 0.14±0.02 | 0.14±0.02 | 0.14±0.01 |
| **Lung** | 0.20±0.03 | 0.20±0.03 | 0.19±0.03 |
| **Kidney** | 0.40±0.04 | 0.38±0.03 | 0.40±0.04 |

Data are expressed as the means ± S.D. (n=10).
