# Supplementary material for: A Water-Soluble Inclusion Complex of Pedunculoside with the Polymer β-Cyclodextrin: A Novel Anti-Inflammation Agent with Low Toxicity
Source: PLoS One. 2014 Jul 11;9(7):e101761. doi: 10.1371/journal.pone.0101761 (PMC4094462; doi:10.1371/journal.pone.0101761)
Supplement: Supplementary file 5 [file pone.0101761.s001.doc]

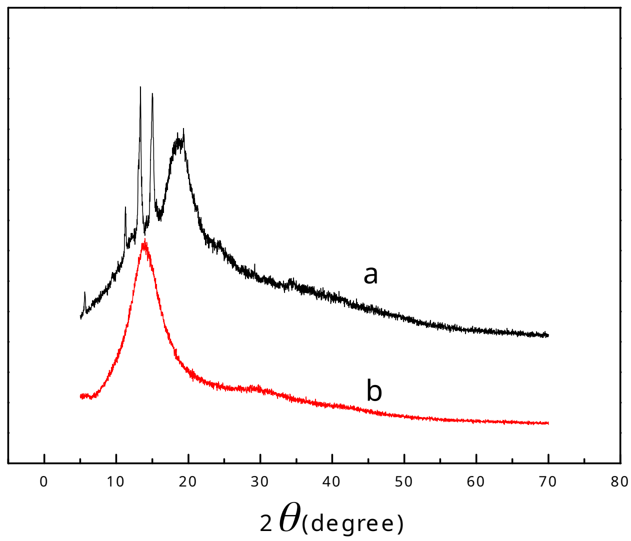

a
b
0
10
20
30
40
50
60
70
80
2θ(degree)
