# Supplementary material for: A Water-Soluble Inclusion Complex of Pedunculoside with the Polymer β-Cyclodextrin: A Novel Anti-Inflammation Agent with Low Toxicity
Source: PLoS One. 2014 Jul 11;9(7):e101761. doi: 10.1371/journal.pone.0101761 (PMC4094462; doi:10.1371/journal.pone.0101761)
Supplement: Supplementary file 6 [file pone.0101761.s002.doc]

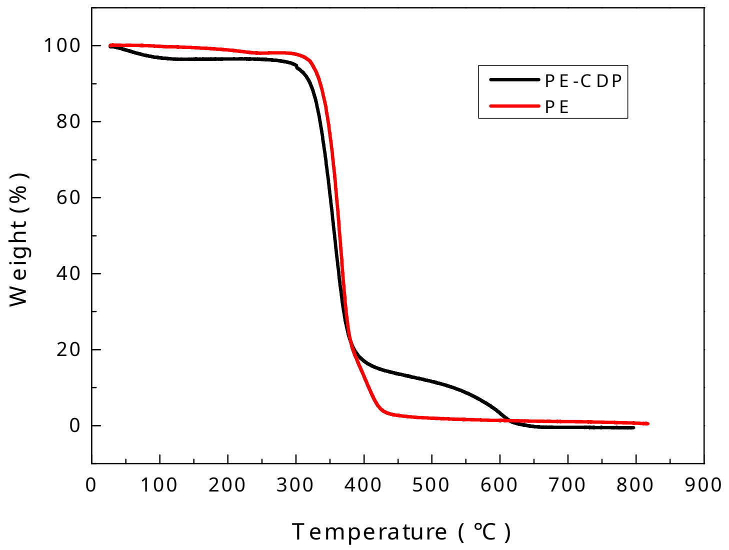

PE-CDP
PE
Weight (%)
Temperature (℃)
0
20
40
60
80
100
0
100
200
300
400
500
600
700
800
900
