# Supplementary material for: A Water-Soluble Inclusion Complex of Pedunculoside with the Polymer β-Cyclodextrin: A Novel Anti-Inflammation Agent with Low Toxicity
Source: PLoS One. 2014 Jul 11;9(7):e101761. doi: 10.1371/journal.pone.0101761 (PMC4094462; doi:10.1371/journal.pone.0101761)
Supplement: Supplementary file 7 [file pone.0101761.s003.doc]

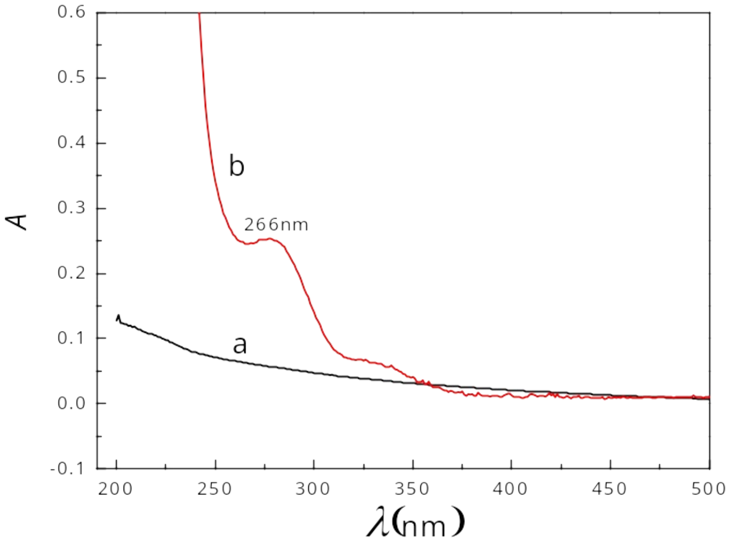

0.6
0.5
0.4
0.3
0.2
0.1
0.0
-0.1
A
b
266nm
a
200
250
300
350
400
450
500
λ(nm)
